# Assessment of the glymphatic dysfunction in amyotrophic lateral sclerosis using the diffusion tensor imaging along the perivascular spaces index: a pilot study

**DOI:** 10.3389/fnagi.2025.1570327

**Published:** 2025-05-13

**Authors:** Seol-Hee Baek, Woo-Suk Tae, Jin-Woo Park, Byung-Jo Kim

**Affiliations:** ^1^Department of Neurology, Korea University Anam Hospital, Korea University College of Medicine, Seoul, Republic of Korea; ^2^Brain Convergence Research Center, Korea University College of Medicine, Seoul, Republic of Korea

**Keywords:** amyotrophic lateral sclerosis, glymphatic system, diffusion tensor imaging, neurodegeneration, biomarker

## Abstract

**Background:**

The glymphatic system plays a critical role in clearing interstitial waste from the brain. Dysfunction of this system has been linked to various neurodegenerative diseases, including amyotrophic lateral sclerosis (ALS). The diffusion tensor imaging-along the perivascular space (DTI-ALPS) index has emerged as a potential neuroimaging biomarker for evaluating glymphatic function. This study investigates whether glymphatic function differs in individuals with ALS compared to those with Parkinson's disease (PD) and normal controls (NCs), using the DTI-ALPS index.

**Methods:**

This study included 35 ALS patients, 35 age- and sex-matched PD patients, and 13 NCs. Diffusion tensor imaging (DTI) was conducted, and the DTI-ALPS index was calculated. Clinical assessments included demographic data, disease duration, cognitive status, and functional scales. Group comparisons and correlation analyses were performed to assess the relationship between the DTI-ALPS index and clinical parameters.

**Results:**

The ALS group exhibited a significantly lower right-side DTI-ALPS index than the NC group (*p* = 0.037), while no differences were observed between the ALS and PD groups. The DTI-ALPS index was negatively correlated with age in ALS and PD groups but showed no correlation with clinical measures in the ALS group. Women in the ALS group had a significantly higher DTI-ALPS index than in men.

**Conclusion:**

Glymphatic dysfunction may contribute to the pathogenesis of ALS, as evidenced by a reduced DTI-ALPS index compared to NCs. However, its clinical relevance and specificity for ALS remain uncertain. Further studies with larger cohorts are warranted to validate these findings.

## 1 Introduction

The glymphatic system is a highly organized brain fluid transport network that facilitates the influx of cerebrospinal fluid (CSF) into the brain parenchyma along the periarterial space and the removal of interstitial fluid (ISF) along the perivenous space (Iliff et al., 2012). This CSF–ISF interchange pathway was first identified in the mouse brain in 2012 (Iliff et al., 2012) and has since been shown to play a role in clearing waste products (Iliff et al., 2012, 2013a). Recently, the glymphatic system has been recognized for its significant role in the pathogenesis of various neurological disorders. Previous studies have reported impaired glymphatic function in mouse models of stroke, Alzheimer's disease, and traumatic brain injury (Arbel-Ornath et al., 2013; Iliff et al., 2014).

Despite the growing recognition of the glymphatic system's role in neurological disorders, assessing its function through neuroimaging remains challenging. Among various imaging techniques, diffusion tensor imaging (DTI) is a valuable tool for investigating microstructural changes in the brain by measuring the movement of water molecules within the tissue. This capability has led to the widespread use of this tool in neurological research (Tae et al., 2018). Recently, the diffusion tensor imaging-analysis along the perivascular space (DTI-ALPS) index has been proposed as an indirect indicator of glymphatic function (Taoka et al., 2017). Numerous studies using the DTI-ALPS index have revealed lower values in various neurological disorders, such as Alzheimer's disease and Parkinson's disease (PD), compared to normal controls (NCs) (Chen et al., 2021; Steward et al., 2021; Bae et al., 2023a). Furthermore, the DTI-ALPS index has been correlated with measures of functional status, such as the Mini-Mental State Examination (MMSE) score and the Unified Parkinson's Disease Rating Scale (UPDRS) score (Chen et al., 2021; Steward et al., 2021; Bae et al., 2023a). These findings suggest that the glymphatic system may be impaired in neurodegenerative diseases, suggesting that the DTI-ALPS index could serve as a useful surrogate marker for evaluating glymphatic function.

Amyotrophic lateral sclerosis (ALS) is a neurodegenerative disease that affects both upper and lower motor neurons. Previous research has shown that pathological aggregation of superoxide dismutase type 1 (SOD1) accumulates in aquaporin-4 (AQP4) knockout mice compared to wild-type mice (Hirose et al., 2021). In addition, a study using dynamic contrast-enhanced MRI demonstrated impaired glymphatic function in the TAR DNA-binding protein 43 (TDP-43) mouse model (Zamani et al., 2022). Recently, another study reported that the DTI-ALPS index was lower in patients with early-stage ALS compared to normal controls (Liu et al., 2024). These findings suggest that glymphatic function may contribute to the pathogenesis of ALS. However, it remains unclear whether glymphatic function is altered in patients with ALS. Furthermore, it is uncertain whether changes in glymphatic function in patients with ALS differ from those in other neurodegenerative diseases, such as Parkinson's disease. Therefore, this study aimed to investigate whether glymphatic function in individuals with ALS differs from that in those with PD and normal controls (NCs), using the DTI-ALPS index.

## 2 Materials and methods

### 2.1 Study population

Patients with clinically probable and definite ALS, diagnosed according to the revised El Escorial criteria (Brooks et al., 2000), and who underwent DTI were prospectively recruited at the neurology clinic of a university-affiliated hospital between January 2017 and June 2022. Patients with ALS aged 51–80 years at the time of DTI were enrolled in this study. The collected demographic and clinical data included sex, age at the time of DTI, age at symptom onset, region of symptom onset, disease duration at the time of DTI, the revised Amyotrophic Lateral Sclerosis Functional Rating Scale (ALSFRS-R) score, delta ALSFRS-R score, MMSE score, and years of education. The delta ALSFRS-R score was calculated using the following equation: (48 - ALSFRS-R score at the time of DTI)/disease duration at the time of DTI (in months).

NCs, who were healthy participants aged 51–80 years with no neurological deficit, were recruited. The collected data from the NCs included demographic and clinical information such as sex and age at the time of the MRI study. In addition, age- and sex-matched patients with PD were enrolled in this study. Data on the patients with PD aged 51–80 years were obtained from the Korea University Anam Hospital Parkinson's registry. Among the registered patients with PD, those who were diagnosed according to the United Kingdom Parkinson's Disease Society Brain Bank Diagnostic Criteria, demonstrated decreased uptake in the basal ganglia on 18F-N-(3-fluoropropyl)-2β-carboxymethoxy-3β-(4-iodophenyl) nortropane (FP-CIT) positron emission tomography (PET), and who underwent DTI were eligible for this study. Each group was matched to the patients with ALS in a 1:1 ratio based on age at the time of DTI and sex. Matching was performed using optimal pair matching with the “*matchit*” package (Stuart et al., 2011) implemented in R. The collected data from the patients with PD included demographic and clinical information such as sex, age at the time of DTI, age at symptom onset, disease duration, Hoehn and Yahr (H&Y) stage, UPDRS Part III score, Montreal Cognitive Assessment (MoCA) score, MMSE score, and years of education.

This study was approved by the Institutional Review Board (IRB) of Korea University Anam Hospital (IRB number: 2015AN0337) and was conducted in accordance with the principles of the Declaration of Helsinki and the relevant institutional guidelines and regulations.

### 2.2 MRI protocol

Imaging data were acquired using a 3T Prisma MRI scanner (Siemens Healthineers, Erlangen, Germany) with a 64-channel head and neck coil. DTI was performed using a single-shot spin-echo-planar imaging pulse sequence with the following parameters: 64 gradients directions plus a B0 image with a b-value = 1,000 s/mm^2^, repetition time/echo time = 3,900–6,500 ms/55 ms, field of view = 224 × 224, matrix = 112 × 112, and slice thickness = 3.0 mm.

### 2.3 MRI preprocessing

Based on a previous study (Liu et al., 2023), a DTI-ALPS pipeline was set up. This pipeline included MRI preprocessing, automatic placement of regions of interest (ROIs), extraction of diffusivity in each axis (x-, y-, and z-axis), and calculation of the DTI-ALPS index. The raw DICOM files were converted to Neuroimaging Informatics Technology Initiative files using the command “*dcm2niix”* (https://github.com/rordenlab/dcm2niix). DTI preprocessing was performed using MRtrix3 (version 3.0.4 Release Candidate 3) (Tournier et al., 2019) and the Functional MRI of the Brain (FMRIB) Software Library (FSL version 6.0.7.11; Oxford, U.K.; http://www.fmrib.ox.ac.uk/fsl/) (Jenkinson et al., 2012). Artifact corrections of DTI imaging were performed using the MRtrix3 command line “*dwidenoise*” for Marchenko-Pastur principal component analysis (MP-PCA) denoising and “*mrdegibbs*” for Gibbs unringing. Eddy current-induced distortions and subject movement were corrected using the FSL's “*eddy*” command. The FA map and x-, y-, and z-axis diffusivity maps were generated using the FSL's “*dtifit*” command line.

### 2.4 Diffusion tensor imaging-along the perivascular space index

**Figure 1:**
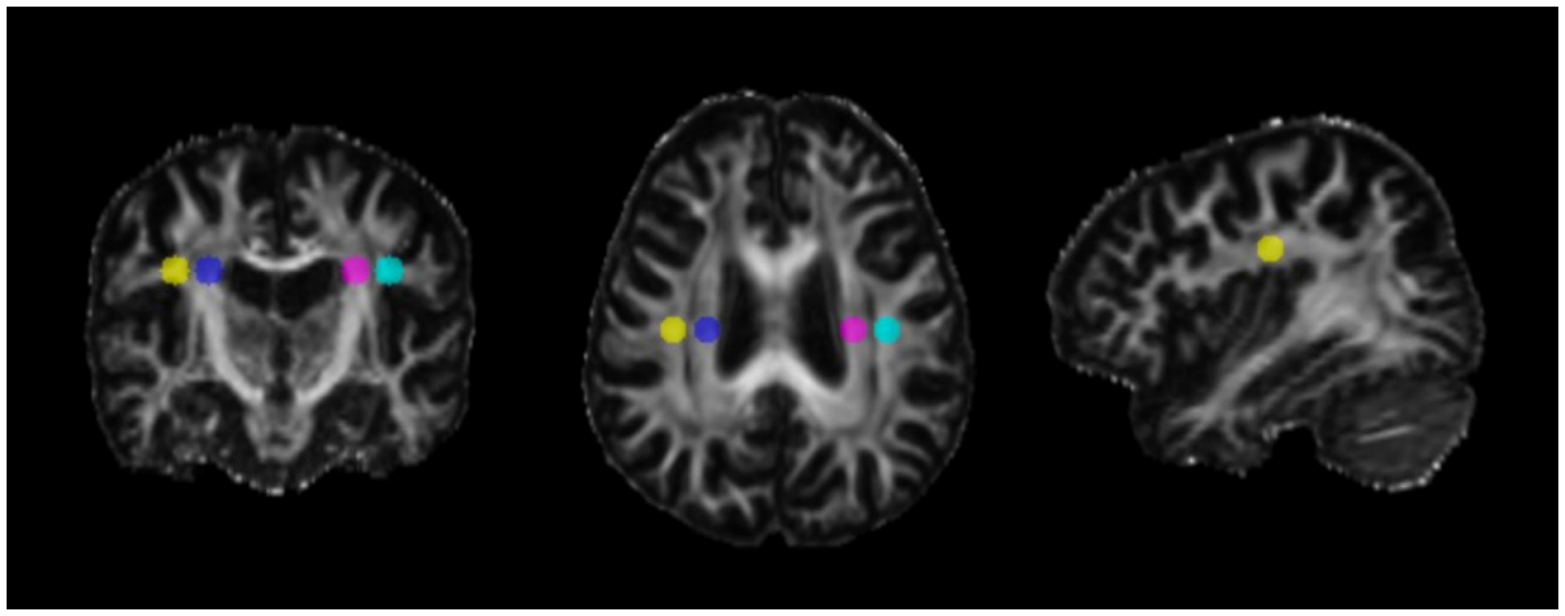
Locations of the regions of interest for the DTI-ALPS index. The regions of interest (ROIs) were demarcated as spheres with a diameter of 5 mm located within the bilateral superior longitudinal fasciculus (SLF) and superior corona radiata (SCR), as depicted in figure. The center coordinates of the ROIs were specified as follows, using the Johns Hopkins University-International Consortium of Brain Mapping-Fractional Anisotropy (JHU-ICBM-FA) template: left SCR (116, 110, 99), left SLF (128, 110, 99), right SCR (64, 110, 99), and right SLF (51, 110, 99). These coordinates were expressed within the voxel space of the FSL's JHU-ICBM-FA-1mm.ni.gz atlas, with the following dimensional ranges: X-axis (right to left), 0 to 181; y-axis (posterior to anterior), 0 to 217; and z-axis (inferior to superior), 0 to 181. Yellow: right superior longitudinal fasciculus, blue: right superior corona radiata, purple: left superior corona radiata, and sky blue: left superior longitudinal fasciculus. The MRI findings are displayed in radiological orientation (Left represents the right side of the brain).

The FA maps for each participant were co-registered to the Johns Hopkins University-International Consortium of Brain Mapping (JHU-ICBM)-FA template using the FSL's “*flirt*” command line. The superior corona radiata (SCR) and superior longitudinal fasciculus (SLF) were identified and labeled as projection and association fibers, according to the JHU-ICBM-DTI-81 White-Matter Labels Atlas. The ROIs were automatically marked as spheres with a 5-mm diameter in the areas of the bilateral SCR and SLF, as depicted in Figure 1. The center coordinates of the ROIs were specified as follows, using the JHU-ICBM-FA template: left SCR (116, 110, 99), left SLF (128, 110, 99), right SCR (64, 110, 99), and right SLF (51, 110, 99). These coordinates were expressed within the voxel space of the FSL's JHU-ICBM-FA-1mm.ni.gz atlas, with the following dimensional ranges: X-axis (right to left), 0 to 181; y-axis (posterior to anterior), 0 to 217; and z-axis (inferior to superior), 0 to 181. The values of the x-axis diffusivity (*Dxxproj*) and y-axis diffusivity (*Dyyproj*) in the projection area and the values of the x-axis diffusivity (*Dxxassoc*) and z-axis diffusivity (*Dzzassoc*) in the association area were extracted. Based on a previous study (Taoka et al., 2017), the DTI-ALPS index was calculated using the following equation:


$$\begin{array}{r} DTI-ALPS\;index\;=\;mean\;\left(Dxxproj,\;Dxxassco\right)\;/\\ mean\;\left(Dyyproj,\;Dzzassoc\right) \end{array}$$

The left and right DTI-ALPS indices were calculated using the x-, y-, and z-axis values from each side, respectively. The mean DTI-ALPS index was calculated as the average of the left and right DTI-ALPS indices.

### 2.5 DTI analysis

The extraction of volumes of interest (VOIs) for the DTI indices was performed according to the ENIGMA-DTI protocol (https://enigma.ini.usc.edu/protocols/dti-protocols). The VOIs were defined using the JHU-ICBM–DTI-81 White-Matter Labels Atlas within the FSL. For each anatomical VOI, individual mean values for FA, RD, MD, and AD were extracted for subsequent statistical analyses. The methods have been described in detail in a previous publication (Baek et al., 2020). Mean FA, RD, MD, and AD values were extracted from the bilateral corticospinal tract (CST) and the posterior limb of the internal capsule (PLIC)—the most affected tracts in ALS—as well as from the bilateral superior longitudinal fasciculus (SLF) and superior corona radiata (SCR), which are associated with the DTI-ALPS index, for further statistical analyses.

### 2.6 Statistical analysis

For clinical data, descriptive summaries were presented as frequency and proportion for categorical variables and as mean (standard deviation, SD) or median (interquartile range, IQR) for continuous variables, depending on data distribution. The data were evaluated for normality using the Shapiro–Wilk test. An independent samples *t*-test or Mann–Whitney U test was used to compare the continuous variables between the two groups. A chi-squared test was used to compare the categorical variables. To evaluate sex differences in the DTI-ALPS index within each group, an independent samples *t*-test or Mann–Whitney U test was performed. In addition, a generalized linear model with age at the time of the study and disease duration as covariates was used to compare the DTI-ALPS index between the male and female participants in each group. A generalized linear model, adjusted for sex and age at the time of the study, was used to compare DTI findings between the ALS and NC groups, as well as between the ALS and PD groups. Pearson's correlation analysis was conducted to examine the relationship between the DTI-ALPS index and age at the time of the study. To adjust for the confounding effect of age, partial correlation analysis was performed with age as a covariate to assess the relationship between the DTI-ALPS index and clinical data, including disease duration, King's stage, ALSFRS-R, delta ALSFRS-R, MMSE score, H&Y stage, UPDRS part III score, and MoCA score. Univariate and multivariate linear regression analyses were performed to examine the correlation between the mean DTI-ALPS index and clinical data in this study group and to identify independent predictive factors of the mean DTI-ALPS index. Variables with a *p*-value < 0.2 in the univariate linear regression analysis were included in the multivariate regression analysis, which was conducted using stepwise regression methods. A *p*-value of < 0.05 was considered statistically significant. All statistical analyses were performed using SPSS (version 20.0).

## 3 Results

### 3.1 Demographic and clinical characteristics of the study population

A total of 35 patients with ALS (mean age 67.40 years), 35 patients with PD (mean age: 67.66 years), and 13 NCs (mean age: 62.62 years) were enrolled in this study. There were no significant differences in age at the time of the study and sex between the ALS, PD, and NC groups. The mean age at symptom onset was 65.80 years in the ALS group and 66.51 years in the PD group. The median disease duration was 18.60 months in the ALS group and 12.27 months in the PD group (*p* = 0.022). In the ALS group, the median ALSFRS-R score at the time of the study was 39.00 (IQR 33.5–42.0) and the median delta ALSFRS-R score was 0.53 (IQR 0.24–1.01). In the PD group, the median UPDRS part III score was 22.0 (IQR 19.0–27.75) and the median H&Y stage was 2.0 (IQR 2.0–2.5). The demographic and clinical data of the study population are summarized in Table 1.

**Table 1:** The demographic and clinical data of the study population.

**Variables**	**ALS**	**PD**	**NC**	***P*** **-value**		
				**ALS vs. PD**	**ALS vs. NC**	**PD vs. NC**
Number of participants	35	35	13			
Sex, male (%)	19 (54.3%)	18 (51.4%)	8 (61.5%)	>0.999	0.750	0.746
Age at study time, years;	67.40 (7.90)	67.66 (6.73)	62.62 (6.33)	0.884	0.056	0.024
Age at symptom onset, years;	65.80 (7.67)	66.51 (7.43)	-	0.693	-	-
Disease duration, months;	18.60 (12.17–36.50)	12.27 (5.87–24.13)	-	0.022	-	-
MMSE score	27.0 (25.0–28.5)	27.0 (25.0–29.0)	-	0.899	-	-
ALSFRS-R score	39.0 (33.5–42.0)					
Delta ALSFRS-R score	0.53 (0.24–1.01)					
King's stage, *n* (%)						
Stage 1	11 (31.4%)					
Stage 2	6 (17.1%)					
Stage 3	16 (45.7%)					
Stage 4a	2 (5.7%)					
Onset region						
Bulbar	10 (28.6%)					
Upper limb	14 (40.0%)					
Lower limb	11 (31.4%)					
Parkinson evaluation						
MOCA	-	24.0 (21.0–28.0)				
UPDRS, part III	-	22.0 (19.0–27.75)				
H & Y stage	-	2.0 (2.0–2.5)				
DTI-ALPS index						
Left side	1.276 (0.139)	1.281 (0.142)	1.321 (0.118)	0.864	0.291	0.363
Right side	1.246 (0.115)	1.275 (0.156)	1.330 (0.135)	0.380	0.037	0.263
Mean	1.261 (0.113)	1.278 (0.140)	1.326 (0.110)	0.571	0.081	0.272

All categorical variables were presented as *n* (%). All continuous variables were presented as mean (standard deviation), except for disease duration, MMSE score, ALSFRS-R score, delta ALSFRS-R score, MOCA, UPDRS part III, and H and Y stage, which were presented as median (IQR). ALS, amyotrophic lateral sclerosis; PD, Parkinson's disease; NC, Normal controls; MMSE, mini-mental status examination; ALSFRS-R, revised Amyotrophic Lateral Sclerosis Functional Rating Scale; MoCA, Montreal Cognitive Assessment; UPDRS, Unified Parkinson's Disease Rating Scale; Hoehn and Yahr (H & Y) stage; DTI-ALPS index, diffusion tensor imaging along the perivascular spaces index.

### 3.2 Comparisons of the DTI-ALPS index

**Figure 2:**
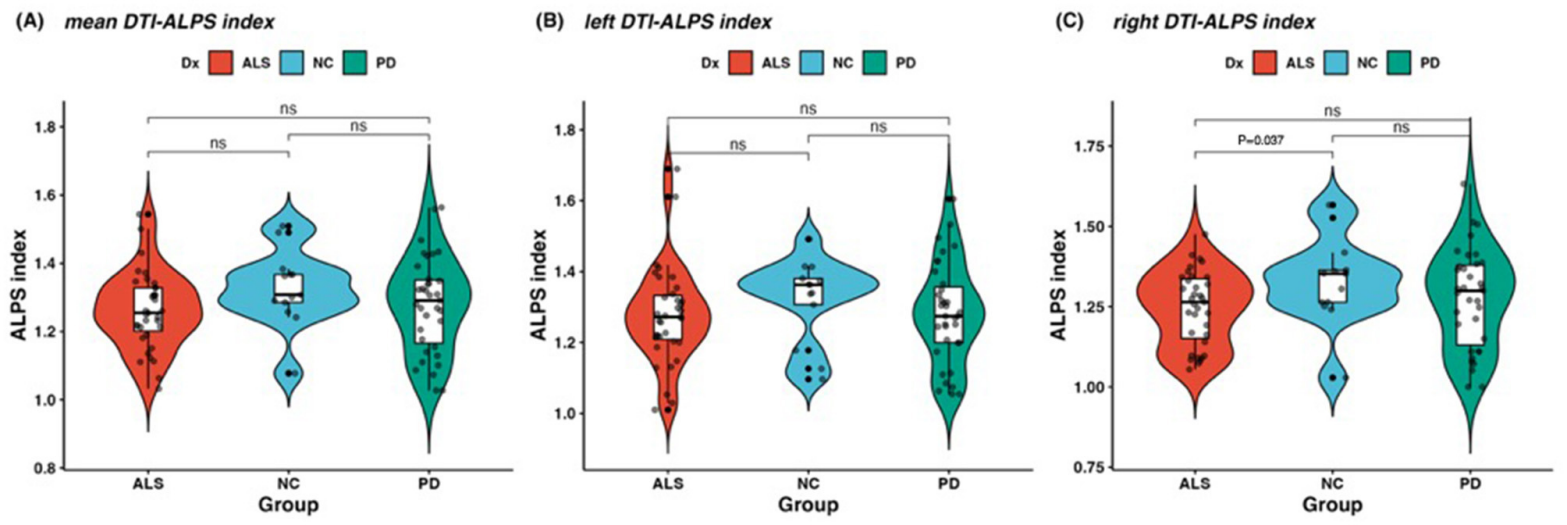
Comparison of the mean, right, and left DTI-ALPS indices among the three groups. **(A)** The mean DTI-ALPS index did not differ significantly between the ALS and PD groups. **(B)** The left-side DTI-ALPS index showed no significant differences between the ALS and PD groups. **(C)** The right-side DTI-ALPS index was lower in the ALS group than in the NC group. No significant differences were found between the ALS and PD groups for any of the DTI-ALPS indices.

The right-side DTI-ALPS index in the ALS group was lower than that in the NC group (*p* = 0.037; Figure 2). However, there was no significant difference in the left-side DTI-ALPS index and the mean DTI-ALPS index between the ALS and NC groups. In addition, there was no significant difference in the right-side, left-side, and mean DTI-ALPS index between the ALS and PD groups. The data on the DTI-ALPS index parameters are summarized in Table 1. The female participants had a higher mean and left-side DTI-ALPS index than the male participants in the ALS group (*p* = 0.004 and *p* = 0.002, respectively; Supplementary Figure). However, there were no significant differences in any of the DTI-ALPS indices between the male and female participants in the PD and NC groups.

### 3.3 Comparison of the DTI values in each VOI

The ALS group showed significantly lower FA values in the right CST (*p* = 0.020) compared to the NC group, while no significant differences were observed in the left CST, bilateral PLIC, SCR, or SLF. MD values were significantly higher in the right PLIC (*p* = 0.040) in the ALS group compared to the NC group, but no differences were observed in other regions. RD and AD values showed no significant differences across all regions. In addition, FA, AD, RD, and MD values did not differ significantly between the ALS and PD groups across all regions. A comparison of the VOIs for each DTI scalar between the two groups is summarized in the Supplementary Table.

### 3.4 Correlation analysis

**Figure 3:**
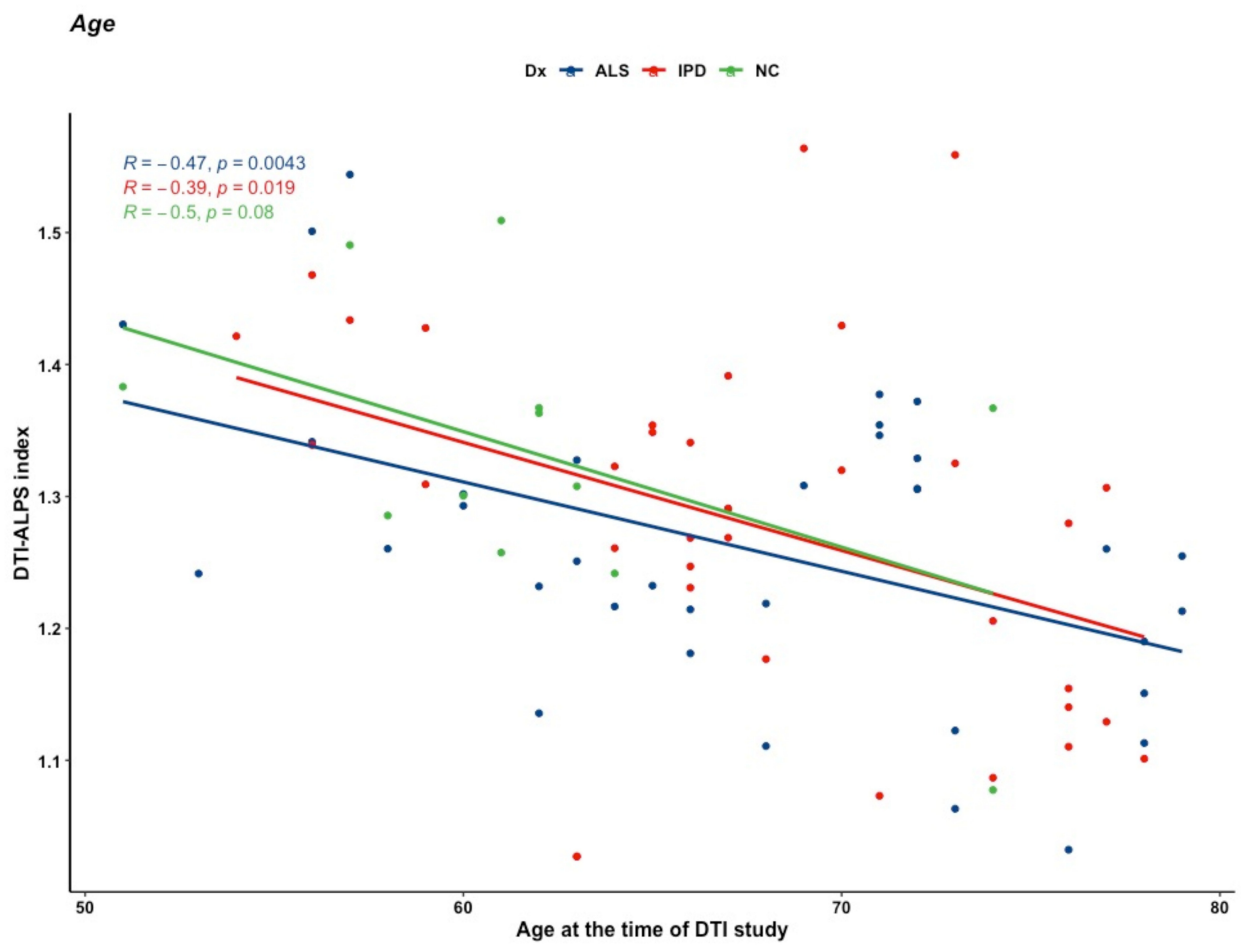
Correlation between the mean DTI-ALPS index and age at the time of DTI. The mean DTI-ALPS index was negatively correlated with age at the time of the study in the ALS (r = −0.471, *p* = 0.004) and PD groups (r = −0.394, *p* = 0.019).

**Figure 4:**
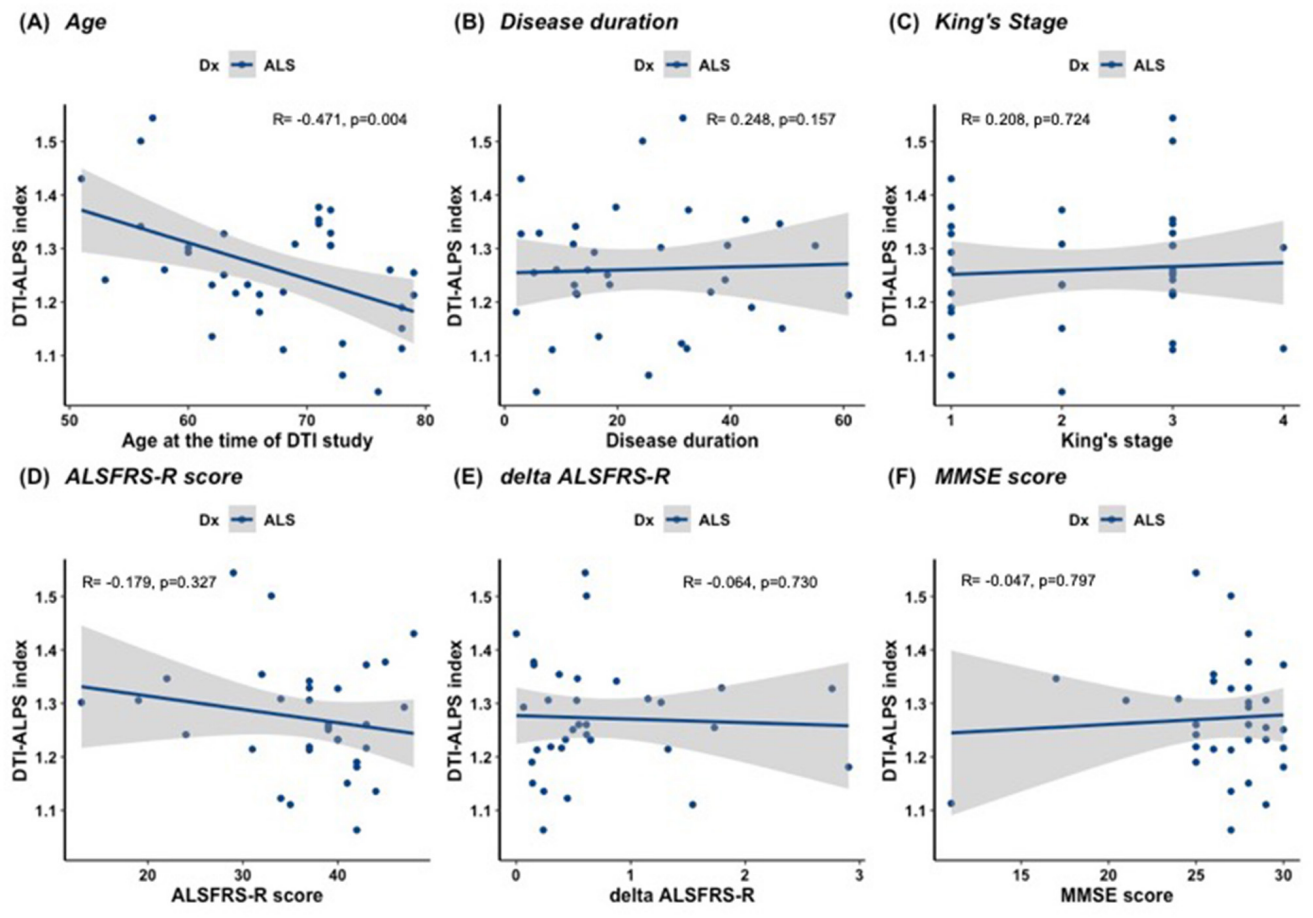
Correlation between the mean DTI-ALPS index and clinical data in ALS. **(A)** The mean DTI-ALPS index was negatively correlated with age at the time of the study in the ALS group. **(B–F)** To adjust for the confounding effect of age, a partial correlation analysis was performed with age as a covariate. There were no significant correlations between the mean DTI-ALPS index and disease duration **(B)**, King's stage **(C)**, ALSFRS-R score **(D)**, delta ALSFRS-R **(E)**, and MMSE score **(F)** in the ALS group.

**Figure 5:**
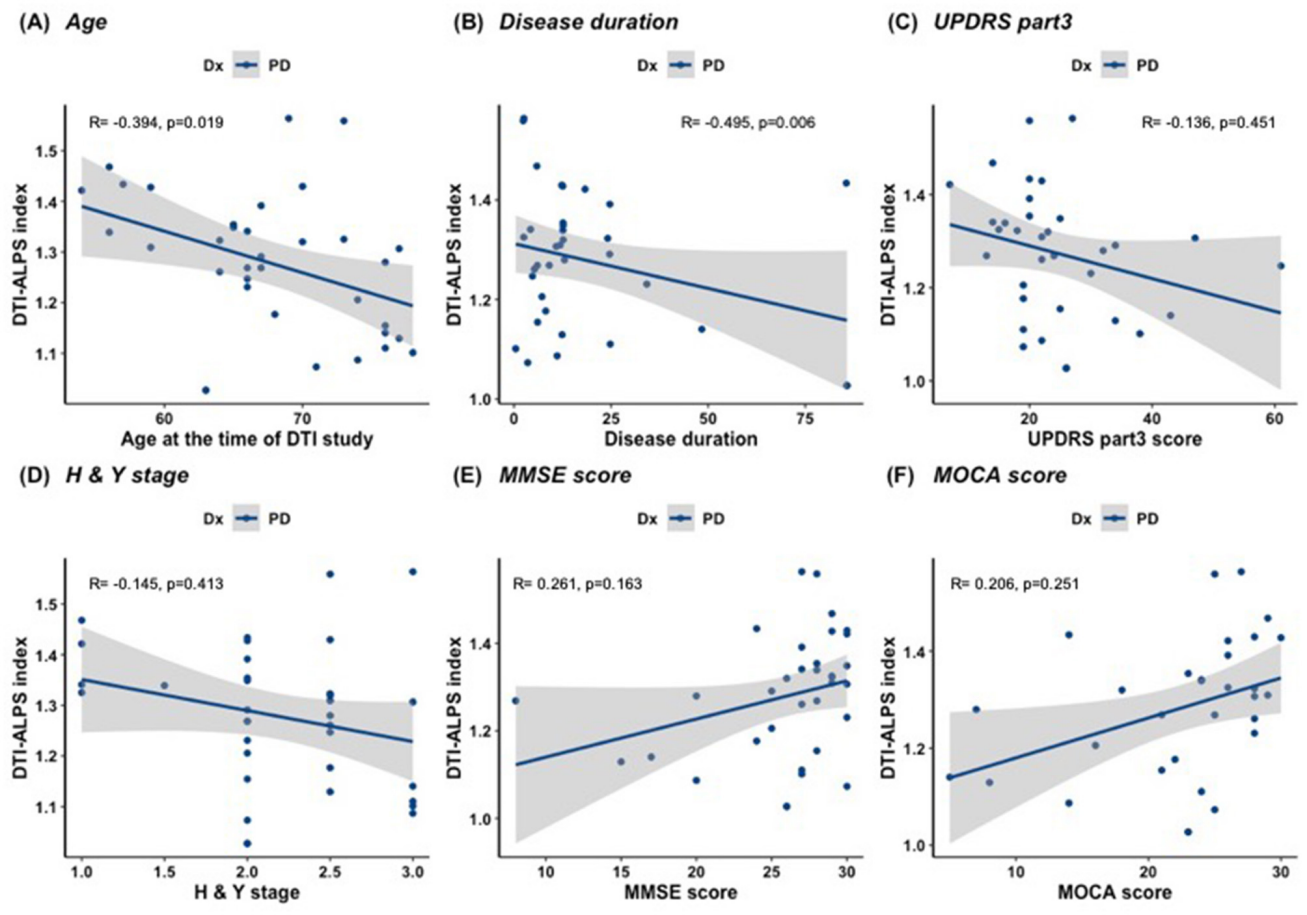
Correlation between the mean DTI-ALPS index and clinical data in PD. **(A)** The mean DTI-ALPS index was negatively correlated with age at the time of the study in the PD group. **(B–F)** To adjust for the confounding effect of age, a partial correlation analysis was performed with age as a covariate. The mean DTI-ALPS index was negatively correlated with disease duration **(B)**. However, there were no significant correlations between the mean DTI-ALPS index and the UPDRS part III score **(C)**, H and Y stage **(D)**, MoCA score **(E)**, and MMSE score **(F)** in the PD group.

A correlation analysis was performed between the DTI-ALPS index and clinical data. The mean DTI-ALPS index was negatively correlated with age at the time of the study in the ALS and PD groups (r = −0.471, *p* = 0.004 and r = −0.394, *p* = 0.019, respectively; Figure 3). To adjust for the confounding effect of age, a partial correlation analysis was subsequently performed with age as a covariate. In the ALS group, the mean DTI-ALPS index showed no significant correlation with disease duration (r = 0.248, *p* = 0.157; Figure 4), King's stage (r = 0.208, *p* = 0.724), ALSFRS-R score (r = −0.179, *p* = 0.327), delta-ALSFRS-R (r = −0.064, *p* = 0.730), or MMSE score (r = −0.047, *p* = 0.797). In the PD group, the mean DTI-ALPS index was negatively correlated with disease duration (r = −0.495, *p* = 0.006; Figure 5). However, there were no significant correlations between the mean DTI-ALPS index and the UPDRS Part 3 score (r = −0.136, *p* = 0.451), H&Y stage (r = −0.145, *p* = 0.413), MMSE score (r = 0.206, *p* = 0.251), or MoCA score (r = 0.261, *p* = 0.163; Figure 5).

### 3.5 Univariate and multivariate regression analysis

According to the univariate analysis, age at the time of DTI (β = −0.008; *p* < 0.001) and sex (female) (β = 0.072; *p* = 0.008) were associated with the mean DTI-ALPS index in this study group. There were no significant associations between the study group (normal controls vs. both ALS and PD groups) and the mean DTI-ALPS index. The multivariate regression analysis revealed that age at the time of DTI (β = −0.007; *p* < 0.001) and female sex (β = 0.065; *p* = 0.008) were significantly associated with the mean DTI-ALPS index in this study group (Table 2).

**Table 2:** Univariate and multivariate linear regression analysis.

**Variable**	**Univariate**		**Multivariate**	
	β	***P*** **-value**	β	***P*** **-value**
Age at the time of the study	−0.008	<0.001	−0.007	<0.001
Sex: female	0.072	0.008	0.064	0.009
Study group: normal control	0.057	0.136	-	-

## 4 Discussion

Our study demonstrated that the right-side DTI-ALPS index was significantly reduced in the ALS group compared to the NC group. However, there was no significant difference in any of the DTI-ALPS indices between the ALS and PD groups. In the ALS group, the left-side, right-side, and mean DTI-ALPS indices were higher in the female participants compared to the male participants. However, no significant difference was observed based on sex in the PD and NC groups. Our study revealed that the DTI-ALPS index was negatively correlated with age at the time of the study in the ALS group. However, there were no significant correlations between the DTI-ALPS index and clinical data, including disease duration, King's stage, ALSFRS-R score, delta ALSFRS-R, and MMSE score, in the ALS group. The results of this study imply that glymphatic function may be impaired in ALS, potentially contributing to the pathogenesis of the disease.

In our study, the ALS group had a lower DTI-ALPS index than the NC group. To the best of our knowledge, only two studies using the DTI-ALPS index in patients with ALS have been reported. One study revealed that the DTI-ALPS index was reduced in the early stage of ALS compared to the NC group (Liu et al., 2024). The other study also showed that the DTI-ALPS index was reduced in the ALS group compared to the primary lateral sclerosis and NC groups (Sharkey et al., 2024). In addition, previous murine studies have demonstrated impaired glymphatic function in ALS mouse models (Hirose et al., 2021; Zamani et al., 2022). All these findings suggest that glymphatic function could be impaired in ALS and may be associated with the pathogenesis of the disease. The currently recognized pathological pathways in ALS include RNA dysregulation, proteostasis impairment, mitochondrial dysfunction, and DNA damage (Goutman et al., 2022; Lim et al., 2023). Impaired glymphatic function may lead to the increased accumulation and decreased elimination of abnormal protein aggregates, thereby potentially contributing to the development of ALS.

Various factors such as aging, sleep, circadian rhythm, and arterial pulsation could influence glymphatic function (Iliff et al., 2013b; Xie et al., 2013; Kress et al., 2014; Hablitz et al., 2020). Interestingly, these factors could contribute to ALS. Aging is a major unmodifiable risk factor for ALS. The incidence of ALS is low before the age of 40, increases between the ages of 70 and 80, and then decreases after the age of 80 (Marin et al., 2018; Jun et al., 2019). In addition, patients with ALS exhibited reduced sleep time, fragmented sleep, decreased sleep efficiency, and increased periodic leg movements during sleep (Lo Coco et al., 2017; Lucia et al., 2021). One study reported that poor sleep quality may be present at the time of ALS diagnosis (Diaz-Abad et al., 2018). A recent study revealed that sleep efficiency and the periodic limb movement index were significantly related to the DTI-ALPS index (Liu et al., 2024). In addition, one study reported that dysfunction of the circadian rhythm was related to disease onset and progression of ALS in a murine model (Huang et al., 2018). All these findings imply that sleep problems could be associated with ALS and may also be related to the impairment of glymphatic function in ALS. In summary, glymphatic function could be impaired in ALS, and it may be associated with the pathogenesis of the disease itself or related clinical characteristics such as old age or sleep problems.

In our study, the DTI-ALPS index was higher in the female patients with ALS compared to the male patients, but no significant differences were observed between the male and female participants in the PD and NC groups. Previous epidemiological studies have consistently shown that the prevalence of ALS is higher in male individuals than in female individuals (Jun et al., 2019; Mehta et al., 2023; Sopranzi et al., 2024). It is possible that sex differences in glymphatic function may contribute to this disparity in the incidence of ALS. It is uncertain whether there are sex differences in glymphatic function. One murine study reported no sex differences in glymphatic influx (Giannetto et al., 2020). However, several human studies using the DTI-ALPS index have shown that glymphatic function varies by sex, with female individuals exhibiting a higher DTI-ALPS index than male individuals. Zhang et al. (2021) reported that female individuals had a higher DTI-ALPS index in the elderly population. Wei et al. (2023) identified sex as one of the unmodifiable factors of the DTI-ALPS index, and a positive correlation was found between female sex and the DTI-ALPS index (standardized mean differences 1.0769). Recently, a large MRI-based study demonstrated that the DTI-ALPS index was higher in female individuals than male individuals (Clark et al., 2024). These findings imply that glymphatic function may differ between sexes in humans. However, it still remains unclear what causes this sex difference in glymphatic function. Previous studies have reported that female individuals may have higher brain metabolism compared to male individuals (Goyal et al., 2019; Baik et al., 2023). This sex difference in brain metabolism could be potentially associated with CSF flow or glymphatic function. Further investigations with larger study populations are needed to clarify this relationship.

Our study revealed no correlation between the DTI-ALPS index and clinical data in ALS, including disease duration, King's stage, ALSFRS-R, delta ALSFRS-R, and MMSE score. In two previously reported studies, the correlation between the DTI-ALPS index and clinical data showed inconsistent results. One study showed that the DTI-ALPS index was significantly correlated with the ALSFRS-R score (Liu et al., 2024), whereas the other study did not observe such a correlation (Sharkey et al., 2024). Although the association between the DTI-ALPS index and clinical status in ALS remains unclear, this inconsistency in results may be related to the fact that ALS is a neurodegenerative disease involving both upper and lower motor neurons. As previously stated, the glymphatic system is the waste clearance system of the brain. Therefore, several studies have demonstrated significant correlations between the DTI-ALPS index and functional status in various brain diseases, including Alzheimer's disease and PD (Chen et al., 2021; Steward et al., 2021; Bae et al., 2023a). However, ALS involves not only brain degeneration but also peripheral nerve degeneration. Therefore, the DTI-ALPS index may be less strongly related to clinical status in ALS compared to other neurodegenerative diseases.

Our study suggests that the DTI-ALPS index has potential as a diagnostic marker for differentiating neurodegenerative diseases from normal controls. However, it did not distinguish between ALS and PD. This may be due to glymphatic dysfunction being a common pathway in the pathogenesis of neurodegenerative diseases, rather than a process specific to each disease. Recently, a meta-analysis reported that the DTI-ALPS index might have potential in distinguishing between Alzheimer's disease and PD (Costa et al., 2024). Future studies are needed to clarify whether the DTI-ALPS index can serve as a useful diagnostic biomarker for not only differentiating normal controls but also for distinguishing among various neurodegenerative diseases.

Our study found a significant decrease in the DTI-ALPS index only in the right hemisphere, with no differences observed in the left hemisphere or the average values. Asymmetry in the DTI-ALPS index has been reported in several studies. Zhao et al. (2023) and Liu et al. (2024) reported a lower index in the ipsilateral hemisphere in patients with temporal lobe epilepsy, while Zhang et al. (2022) found a lower index on the lesion side in patients with spontaneous intracerebral hemorrhage. Similarly, Shen et al. (2022) observed asymmetry in Parkinson's disease, with a lower index in the left hemisphere during the early stages, which later affected both hemispheres in the advanced stages. ALS is characterized by gradually progressive, asymmetric motor weakness, which may be related to asymmetry in the DTI-ALPS index. However, whether patients with ALS exhibit differences in the DTI-ALPS index between the right and left hemispheres remains unclear, as all previous studies in ALS have used the average values of ROIs in both hemispheres. Further research is needed to clarify this issue.

Our study has some limitations. First, the study population was relatively small. We observed a significantly lower DTI-ALPS index in the ALS and PD groups compared to the NC group, as well as a sex difference in glymphatic function in ALS. Further studies with a larger study population are needed to validate these findings. Second, our study lacked sleep data, which is one of the key factors influencing glymphatic function. Previous studies have reported a lower DTI-ALPS index in individuals with sleep disruption, obstructive sleep apnea, restless leg syndrome, and REM sleep behavior disorder (Lee et al., 2022; Bae et al., 2023b; Park et al., 2023; Saito et al., 2023). In addition, the DTI-ALPS index was positively correlated with N2 sleep duration (Siow et al., 2022). Previous studies have reported that patients with ALS frequently experience sleep problems (Lo Coco et al., 2017; Diaz-Abad et al., 2018; Lucia et al., 2021), suggesting that sleep disturbance may contribute to altered glymphatic function in ALS. One study reported that sleep efficiency and the periodic limb movement index were significant predictors of the DTI-ALPS index in early-stage ALS (Liu et al., 2024). Further studies are needed to determine whether glymphatic function varies with the presence or absence of sleep disturbances in ALS. Third, although the DTI-ALPS index is considered an indirect indicator of glymphatic function, its fundamental methodology raises concerns about its ability to accurately assess glymphatic function (Ringstad, 2024; Taoka et al., 2024). One major issue is that glymphatic clearance (CSF–ISF exchange) plays only a minimal role in the deep white matter, which is the main region of interest in the DTI-ALPS index. In addition, the limited DTI measurement in the white matter provides limited insight into the brain's overall clearance capacity. Moreover, since the perivascular space in the white matter comprises only approximately 1% of the total brain volume, the DTI-ALPS index faces challenges in differentiating perivascular water diffusivity from other forms of directional water movement. Furthermore, although numerous studies have reported an association between the DTI-ALPS index and neurological diseases, correlation does not equate to causation. Consequently, the reliability of the DTI-ALPS index as an indicator of glymphatic brain clearance remains uncertain. Therefore, these limitations should be considered when interpreting the DTI-ALPS index results.

In conclusion, glymphatic dysfunction may be associated with the pathogenesis of ALS. However, there was no difference in the DTI-ALPS index between ALS and PD, and there was no change related to disease progression or clinical characteristics. Although the DTI-ALPS index has potential as a clinical biomarker for not only evaluating glymphatic function but also for differentiating neurodegenerative diseases from normal individuals, its clinical implications remain limited. Further studies with a larger study population are needed to clarify these issues.

## Data Availability

The original contributions presented in the study are included in the article/supplementary material, further inquiries can be directed to the corresponding author/s.
