# Supplementary material for: Assessment of the glymphatic dysfunction in amyotrophic lateral sclerosis using the diffusion tensor imaging along the perivascular spaces index: a pilot study
Source: Front Aging Neurosci. 2025 May 13;17:1570327. doi: 10.3389/fnagi.2025.1570327 (PMC12106509; doi:10.3389/fnagi.2025.1570327)
Supplement: Supplementary file 1 [file Data_Sheet_1.pdf]

Supplementary Figure 1.

1.1 Comparison of DTI-ALPS index between men and women

Women had a higher mean, and left-side DTI-ALPS index than men in the ALS group (p=0.004, and p=0.002, respectively). However, there were no significant differences in the right-side DTI-ALPS index (p=0.068) between men and women, respectively. A generalized linear model, with age at the time of DTI study and disease duration as covariates, demonstrated that women had a higher mean DTI-ALPS index (p=0.001; A), left-side DTI-ALPS index (p=0.001; D), right-side DTI-ALPS index (p=0.043; G) compared to men within the ALS group. However, there was no significant difference in each DTI-ALPS index between men and women in both IPD (B, E, H) and NC (C, F, I) groups.

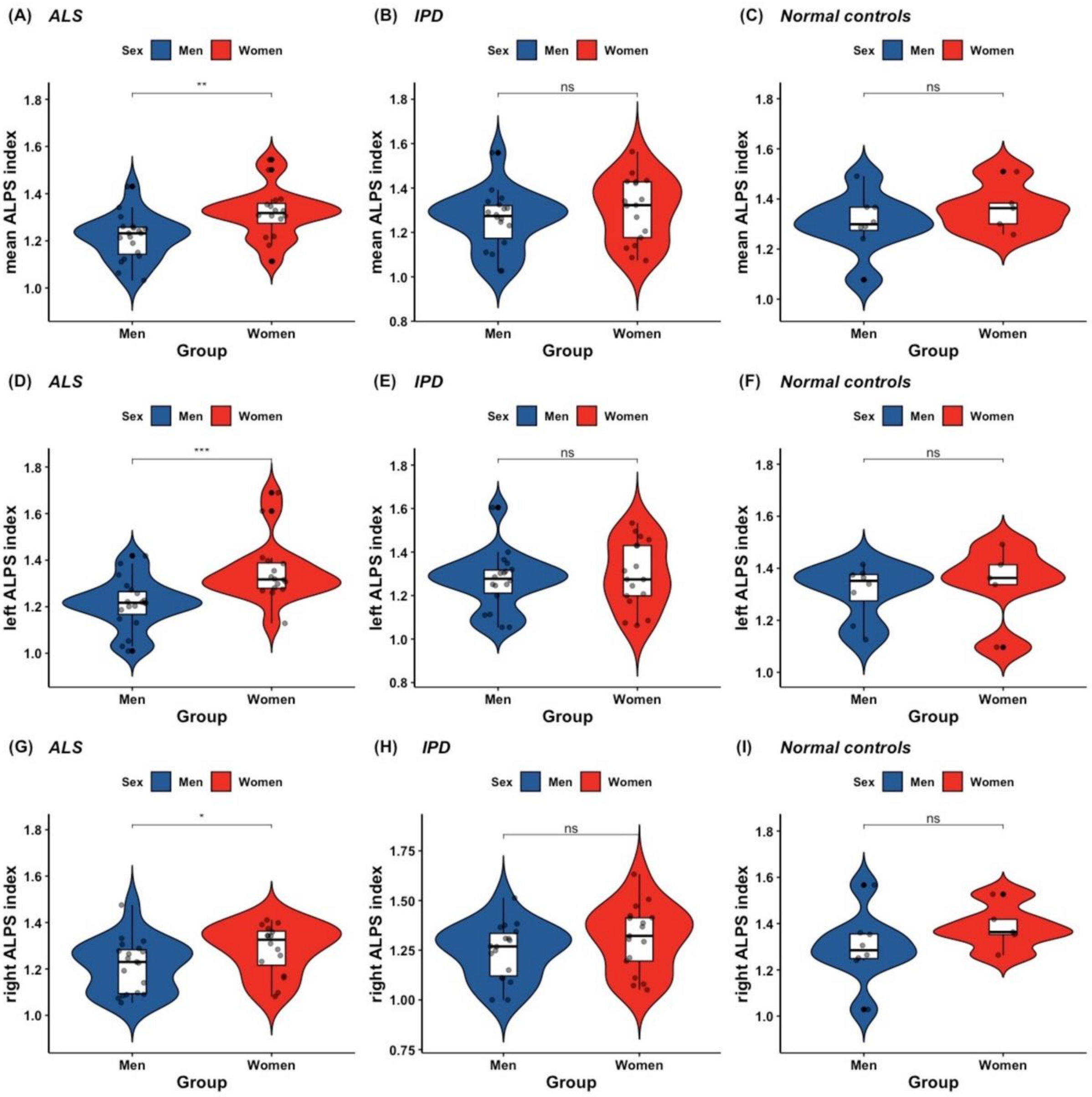

(A) ALS
(B) IPD
(C) Normal controls
(D) ALS
(E) IPD
(F) Normal controls
(G) ALS
(H) IPD
(I) Normal controls
Sex Men Women
mean ALPS index
left ALPS index
right ALPS index
Group
Men
Women
**
***
*
ns

Supplementary Table 1. Comparison of DTI values in each ROI between ALS and NC groups

	FA			RD			MD			AD		
	**ALS**	**NC**	**P-value**	**ALS**	**NC**	**P-value**	**ALS**	**NC**	**P-value**	**ALS**	**NC**	**P-value**
**L CST**	0.587087 (0.029598)	0.590420 (0.043959)	.820	0.000442 (0.000031)	0.000442 (0.000035)	.901	0.000707 (0.000028)	0.000710 (0.000025)	.684	0.001238 (0.000061)	0.001245 (0.000073)	.695
**L PLIC**	0.696202 (0.029549)	0.688654 (0.033501)	.660	0.000369 (0.000028)	0.000371 (0.000029)	.855	0.000718 (0.000017)	0.000710 (0.000012)	.551	0.001416 (0.000044)	0.001389 (0.000051)	.370
**L SCR**	0.526613 (0.028636)	0.525354 (0.030501)	.984	0.000496 (0.000035)	0.000488 (0.000025)	.769	0.000738 (0.000035)	0.000724 (0.000026)	.610	0.001224 (0.000055)	0.001196 (0.000063)	.585
**L SFL**	0.472895 (0.026077)	0.467508 (0.023339)	.396	0.000533 (0.000039)	0.000529 (0.000030)	.833	0.000741 (0.000037)	0.000732 (0.000027)	.865	0.001157 (0.000041)	0.001138 (0.000036)	.398
**R CST**	**0.597255 (0.028858)**	**0.620735 (0.030204)**	**.020**	0.000428 (0.000025)	0.000417 (0.000030)	.197	0.000697 (0.000024)	0.000702 (0.000027)	.570	0.001234 (0.000059)	0.001272 (0.000062)	.071
**R PLIC**	0.704921 (0.024280)	0.710761 (0.032272)	.364	0.000364 (0.000022)	0.000351 (0.000031)	.131	**0.000721 (0.000018)**	**0.000704 (0.000018)**	**.040**	0.001434 (0.000052)	0.001410 (0.000051)	.503
**R SCR**	0.533297 (0.030172)	0.532777 (0.026087)	.937	0.000490 (0.000034)	0.000472 (0.000020)	.255	0.000740 (0.000036)	0.000711 (0.000021)	.068	0.001241 (0.000065)	0.001189 (0.000057)	.108
**R SFL**	0.499560 (0.025909)	0.499857 (0.021081)	.701	0.000520 (0.000037)	0.000507 (0.000025)	.610	0.000748 (0.000035)	0.000729 (0.000025)	.324	0.001203 (0.000046)	0.001172 (0.000037)	.162

All continuous variables were presented as mean (standard deviation).

Abbreviation: FA, fraction anisotropy; RD, radial diffusivity; MD, mean diffusivity; AD, axial diffusivity, ALS, amyotrophic lateral sclerosis, NC, normal controls; L, left; R, right; CST, corticospinal tract; PLIC, posterior limb of internal capsule; SCR, superior corona radiata; SLF, superior longitudinal fasciculus

Supplementary Table 2. Comparison of DTI values in each ROI between ALS and PD groups

	FA			RD			MD			AD		
	**ALS**	**PD**	**P-value**	**ALS**	**PD**	**P-value**	**ALS**	**PD**	**P-value**	**ALS**	**PD**	**P-value**
L CST	0.587087 (0.029598)	0.577945 (0.033947)	.259	0.000442 (0.000031)	0.000448 (0.000032)	.475	0.000707 (0.000028)	0.000705 (0.000023)	.630	0.001238 (0.000061)	0.001219 (0.000049)	.150
L PLIC	0.696202 (0.029549)	0.700878 (0.025455)	.483	0.000369 (0.000028)	0.000365 (0.000028)	.465	0.000718 (0.000017)	0.000717 (0.000024)	.693	0.001416 (0.000044)	0.001422 (0.000045)	.612
L SCR	0.526613 (0.028636)	0.529121 (0.033038)	.713	0.000496 (0.000035)	0.000503 (0.000056)	.597	0.000738 (0.000035)	0.000750 (0.000058)	.364	0.001224 (0.000055)	0.001244 (0.000077)	.218
L SFL	0.472895 (0.026077)	0.476555 (0.039965)	.598	0.000533 (0.000039)	0.000543 (0.000078)	.558	0.000741 (0.000037)	0.000756 (0.000075)	.306	0.001157 (0.000041)	0.001184 (0.000075)	.072
R CST	0.597255 (0.028858)	0.604234 (0.030164)	.309	0.000428 (0.000025)	0.000423 (0.000032)	.449	0.000697 (0.000024)	0.000694 (0.000029)	.632	0.001234 (0.000059)	0.001235 (0.000053)	.916
R PLIC	0.704921 (0.024280)	0.715743 (0.022142)	.060	0.000364 (0.000022)	0.000354 (0.000025)	.070	0.000721 (0.000018)	0.000719 (0.000023)	.601	0.001434 (0.000052)	0.001449 (0.000045)	.196
R SCR	0.533297 (0.030172)	0.536483 (0.028729)	.648	0.000490 (0.000034)	0.000493 (0.000058)	.837	0.000740 (0.000036)	0.000747 (0.000063)	.654	0.001241 (0.000065)	0.001254 (0.000081)	.502
R SFL	0.499560 (0.025909)	0.500987 (0.044011)	.795	0.000520 (0.000037)	0.000531 (0.000085)	.512	0.000748 (0.000035)	0.000764 (0.000080)	.310	0.001203 (0.000046)	0.001228 (0.000081)	.120

All continuous variables were presented as mean (standard deviation).

Abbreviation: FA, fraction anisotropy; RD, radial diffusivity; MD, mean diffusivity; AD, axial diffusivity, ALS, amyotrophic lateral sclerosis, PD, Parkinson's disease; L, left; R, Right; CST, corticospinal tract; PLIC, posterior limb of internal capsule; SCR, superior corona radiata; SLF, superior longitudinal fasciculus
